# Associations between Stigma, Depression, and Adherence to Antiretroviral Therapy in Brazilian Men Who Have Sex with Men Living with HIV

**DOI:** 10.3390/ejihpe14060098

**Published:** 2024-05-23

**Authors:** Felipe Alckmin-Carvalho, Henrique Pereira, António Oliveira, Lucia Nichiata

**Affiliations:** 1 School of Nursing, University of São Paulo, São Paulo 01239-020, Brazil; izumi@usp.br; 2 Department of Psychology and Education, Faculty of Social and Human Sciences, University of Beira Interior, Pólo IV, 6200-209 Covilhã, Portugal; hpereira@ubi.pt (H.P.); antonio.oliveira@ubi.pt (A.O.); 3 Research Center in Sports Sciences, Health Sciences and Human Development (CIDESD), 5001-801 Vila Real, Portugal

**Keywords:** HIV/AIDS, sexual and gender minorities, medication adherence, depression, HIV-related stigma, HIV stigma

## Abstract

Adherence to antiretroviral therapy (ART) is a complex and multi-determined process that is influenced by psychosocial variables. Although international studies have pointed to the adverse impact of HIV stigma, sexual stigma, and depression on ART adherence among men who have sex with men (MSM) with HIV, less is known about this association among Brazilians. We aimed to (a) evaluate indicators of depression, stigma related to HIV and homosexuality, and adherence to ART in a sample of Brazilian MSM living with HIV; (b) assess possible correlations between the variables analyzed, and (c) assess the impact of HIV and sexual stigma and depression on ART adherence. This cross-sectional study comprised 138 Brazilian MSM living with HIV as participants. Scales used included: a sociodemographic/clinical questionnaire, the questionnaire for assessment of adherence to antiretroviral therapy (CEAT-HIV), the Beck depression inventory (BDI-II), the internalized homophobia scale, and the HIV stigmatization scale. The mean adherence score was relatively high (78.83, within a range of 17–89 points). However, we observed inadequate ART adherence (CEAT-HIV < 75) in 28 (20.2%) respondents. Participants reported high scores for internalized sexual stigma, perceived sexual stigma in the community, and HIV stigma. Symptoms of depression were identified in 48.47% of participants. We found negative correlations between depression, HIV stigma, and treatment adherence, but not between sexual stigma and ART adherence. HIV-related stigma and sexual stigma were positively correlated with depression. Our regression analysis indicated that each year of age at diagnosis of HIV increased adherence by 0.22 points, on average. Each additional BDI-II score reduced adherence to ART by 0.20 points. The high prevalence of depression, HIV stigma, and sexual stigma, and their adverse effects on ART adherence and mental health, point to the need to implement evidence-based interventions to reduce sexual and serological stigma in the general population, as well as to mitigate the negative impacts of stigma on MSM living in HIV in Brazil. They also highlight the importance of periodically screening for these variables among MSM treated in Brazilian public health services, especially among those with inadequate adherence to ART.

## 1. Introduction

Acquired immunodeficiency syndrome (AIDS) is a disease characterized as the late manifestation of HIV infection [1]. This condition is an advanced phase of infection in which the virus damages the immune system of the infected individual to such an extent that opportunistic diseases threaten the continuity of life [1]. The HIV/AIDS epidemic, which began in the 1980s, is estimated to be responsible for approximately 40.4 million deaths worldwide as of 2023 [2].

In 2023, international guidelines were proposed to control the HIV/AIDS epidemic by 2030. Their aim was to identify 95% of HIV cases, ensuring optimal treatment adherence for 95% of those identified, and ensuring that 95% of those living with HIV have an undetectable viral load [2]. Studies indicate that controlling HIV and AIDS is a health challenge, particularly among men who have sex with men (MSM). This group has been disproportionately affected by the infection, with an increasing incidence of HIV infection that is also higher than that observed in the general population [3,4,5,6].

In recent decades, global efforts have been made to address HIV/AIDS, and remarkable progress has been achieved. Highly effective and low toxicity antiretrovirals with few adverse effects have been developed. Thus, the physical complications associated with HIV have decreased, and the quality of life and life expectancy of individuals living with the virus have increased considerably [7,8,9]. Currently, the treatment of HIV infection with highly effective antiretrovirals allows the HIV viral load in the infected individual’s blood plasma to drop to undetectable levels within a few months in most cases. Undetectable viral load is associated with a reduction in persistent immunological activation, which considerably reduces the state of chronic inflammation and its long-term risks for the organism, such as cardiovascular, metabolic, and cognitive problems [10,11]. Furthermore, sufficient evidence indicates that people with an undetectable viral load do not transmit the HIV infection sexually, even without using preventive methods [12]. This has profoundly improved the affective sexual lives of people living with HIV [13].

Adherence to medication for chronic diseases is a complex and multifaceted phenomenon. Studies indicate that the leading cause of antiretroviral therapy (ART) failure is poor patient adherence [14,15]. Sociodemographic and clinical variables, such as income, access to health services, number of pills, and adverse effects influence adherence to ART [16,17,18,19]. Furthermore, since the 2000s, studies have reported that psychopathologies, such as depression and anxiety, which either existed before or occurred in reaction to an HIV diagnosis had a harmful effect on ART [20,21]. Recent studies have highlighted the impact of psychosocial variables, such as the quality of interaction with healthcare providers, family and partner support, self-care repertoires, self-efficacy, and coping skills on adherence to ART [22,23,24]. 

Specifically, among MSM living with HIV, studies suggest that HIV-related stigma appears to influence adherence to ART [14,25,26]. Furthermore, perceived sexual stigma in the community and internalized sexual stigma seem to harm ART adherence, although there is no consensus on this relationship. For example, Ortiz-Hernández et al. (2021) [27] evaluated 340 MSM living with HIV in Mexico and found that sexual stigma experienced in the form of violence or discrimination increased the risk of inadequate ART adherence. However, internalized sexual stigma was not associated with ART adherence in this study. The authors argue that, owing to recent cultural, institutional, and legal changes, the expression of sexual stigma may have become more subtle, and older instruments may fail to detect less evident expressions of this phenomenon. However, Johnson et al. (2008) [28] evaluated 465 MSM with HIV and found an indirect relationship between internalized sexual stigma and inadequate adherence to ART. This association was mediated by negative affect and a higher prevalence of substance abuse.

Brazilian studies have aimed to evaluate sociodemographic and clinical variables associated with ART adherence [17,20,29,30]. However, few have investigated the impact of psychopathology and stigma on ART adherence in the general population. To our knowledge, this study is the first to evaluate the impact of psychosocial variables on ART adherence in a sample composed exclusively of Brazilian MSM living with HIV. Considering the specificities of the HIV/AIDS epidemic among MSM living with HIV and the epidemiological importance of this key population in confronting the HIV/AIDS epidemic [3,5], studies evaluating the effects of psychopathology and different types of stigma on ART adherence are needed.

The aims of this study are: (a) to evaluate indicators of depression, stigma related to HIV and homosexuality, and adherence to ART in a sample of Brazilian MSM living with HIV; (b) to assess possible correlations between the variables analyzed; and (c) to assess the impact of HIV-related stigma, sexual stigma, and depression on ART adherence. We believe that we will find high scores of depression and stigma related to HIV and homosexuality, and relatively high rates of adherence to ART, and that there will be negative and significant correlations between depression, sexual, and HIV-related stigma and adherence to ART. Finally, we believe that the negative effects of depression and the forms of stigma investigated will impair adherence to ART.

## 2. Materials and Methods

This is a cross-sectional study and comprised a non-probabilistic sample of Brazilian MSM living with HIV. The snowball method [31] was used to determine the sample composition. This method is useful because the data collection involves a sensitive and intimate topic regarding the disclosure of HIV diagnoses and mental health variables, and people may be less likely to come forward without the recommendation of others who have participated in the study.

The first author contacted two nurses attending master’s and doctoral programs in the School of Nursing at the University of São Paulo to identify the first five seed participants. Another five seed participants were recruited from social networks, such as Instagram and Facebook, on support pages or meeting pages for HIV-positive MSM. At the end of the online form, seed participants were asked, “Do you have any friends, gay or bisexual, who live with HIV, which would be willing to participate in the research?” The following message was displayed: “If so, could you please ask your friend for permission to share the preferred contact method (e-mail, telephone, or social network) so that I can present the survey?”

The inclusion criteria were as follows: being male and having sex with other men, over 18 years old, diagnosed with HIV or AIDS, taking antiretroviral drugs for at least three months (criterion for the application of the adherence assessment instrument), internet access, and being able to privately complete the assessment instruments. The researcher interviewed participants to determine whether they met the inclusion criteria. All participants indicated by the seeds met the inclusion criteria and agreed to complete the evaluation instruments. Participants were selected during the month of August 2021. No saturation criteria associated with participant characteristics were adopted. The possible biases related to the sample composition method are presented in the Section 4.

### 2.1. Instruments

The Questionnaire for the Assessment of Adherence to Antiretroviral Therapy (CEAT-HIV) [30]: This self-report questionnaire is designed for adults living with HIV and receiving ART. The CEAT-HIV is a quick and easy-to-complete tool comprising 20 items covering the primary factors associated with ART adherence behavior, such as frequency of taking medication, understanding the treatment and its effects, and the quality of the relationship with the health team. Scoring was performed using a five-point Likert scale; the higher the score, the higher the degree of treatment adherence. Scores above 74 indicated adequate adherence, and scores of 74 or less indicated inadequate adherence [30]. The instrument has good internal validity (Cronbach’s alpha = 0.71), with evidence of its validity in the Brazilian population [32].

The HIV Stigmatization Scale: This scale comprised 40 items distributed across four subscales: (1) personalized stigmatization, (2) disclosure, (3) negative self-image, and (4) public attitudes [33]. Scoring uses a four-point Likert scale. The overall stigmatization index was obtained from the arithmetic means of the responses. The higher the score, the higher the level of serological stigma. There was no cutoff point for classifying the level of serological stigma. The scale’s internal consistency was considered satisfactory (Cronbach’s alpha = 0.906) [34]. There is evidence of its validity in the Brazilian population [34].

The Internalized Sexual Stigma Scale: This questionnaire evaluates two dimensions—internal and external perception of stigma [35]. All items were written in the affirmative form and measured on a five-point Likert scale ranging from 1 (strongly disagree) to 5 (strongly agree). Examples of the statements are as follows: (1) Typically, effeminate gay men make me feel uncomfortable; (2) I prefer to have anonymous sexual partners; and (3) Life would be easier if I were heterosexual. Higher scores indicated higher levels of internalized sexual stigma. No cut-off point exists for the classification of sexual stigma. In the present study, we used the 19-item Brazilian version of the scale, which showed better internal validity in a validation article [36], with a Cronbach’s alpha of 0.814 for the internal perception of stigma and 0.622 for the external perception of stigma.

The Beck Depression Inventory (BDI-II): This consists of 21 items, each with four alternatives [37]. The questions encompass physical symptoms, such as fatigue, sleep, weight alterations, and cognitive alterations, which occur in patients diagnosed with depression, such as persistent sadness, pessimism, feelings of failure, dissatisfaction, and guilt. Depression levels were classified according to the total score: 0–11, minimal; 12–19, mild; 20–35, moderate; and 36–63, severe. In a validation study of the instrument in a Brazilian population, Cronbach’s alpha was 0.81 [38]. 

The Sociodemographic and Clinical Questionnaire: Developed by the first author, this questionnaire assesses sociodemographic characteristics (age, sex, marital status, education, occupation, and income) and clinical characteristics (age at diagnosis, age at ART initiation, current viral load, and antiretroviral therapy type).

### 2.2. Data Analysis

Statistical analyses were performed using SPSS 20.0, and significance was set at 5% (*p* < 0.05). Descriptive analyses are presented as frequencies, proportions, means, medians, and standard deviations. Data normality was assessed using the Shapiro–Wilk test, and homogeneity of variance was assessed using Levene’s test. Correlation analyses were performed between depression, sexual stigma, HIV-related sigma, and adherence to ART using Pearson’s r-test. A stepwise method was used for the multiple linear regression analysis to evaluate the predictive power of the variables analyzed for treatment adherence. The following variables were tested in linear regression: current age, age at HIV diagnosis, income, education, depression, total sexual stigma, and total HIV stigma.

Cohen’s effect size was used for parametric tests, rank-biserial correlation for nonparametric tests, and phi for categorical tests. Conventionally, a value of d = 0.20 representing a magnitude with a small effect, d = 0.50 indicating a magnitude with a medium effect, and d = 0.80 indicating a magnitude with a high effect was used for all effect sizes [39]. The following intervals were adopted to classify the intensity of the correlation between the variables analyzed: From 0 to 0.30, slight correlation; from 0.30 to 0.70, moderate correlation; and from 0.7 to 1, strong correlation between variables [39].

### 2.3. Ethical Considerations

This study was approved by the Research Ethics Committee of the School of Nursing, University of São Paulo (number: 4.601.952, CAAE: 31527820.7.0000.5392; 19 March 2021). All the participants provided written informed consent.

## 3. Results

In total, 138 Brazilian gay men with HIV participated in this study. The participants’ average age was 36.12 years (SD = 9.03), ranging from 20 to 64 years. Slightly more than half of the participants lived in the city of São Paulo. Average income was 3.72 Brazilian minimum wages, corresponding to R$4508.64 (+/−850 EUR/1.060 $USD) in 2022, the year of data collection, placing participants in the middle-class category (B and C, on a scale from A to E). The average age at HIV diagnosis was 28.80 (SD = 6.84), with a minimum of 24 years and a maximum of 54 years. More than half the participants had completed higher education or postgraduate studies (n = 81, 58.7%). The majority were employed (n = 108; 78.26%), lived in their own homes (n = 64; 43.8%), and often had family members or partners (n = 80; 57.97%). Approximately 90% of the participants reported having an undetectable viral load in their last blood test, and the majority used a combination of Dolutegravir, Tenofovir Fumarate, and Lamivudine as a drug treatment regimen for HIV. Categorical sociodemographic and clinical data of the participants are presented in Table 1.

The average adherence-to-ART score was 78.83 (SD = 6.63), with a minimum score of 46 and a maximum score of 89 (possible range: 17–89). Adherence to ART was inadequate in 28 (20.2%) participants (value < 75 points). Signs and symptoms of depression were identified in 48.47% of the participants: 23.92% mild, 20.22% moderate, and 4.34% severe. The mean depression score was 10.99 (SD = 8.95, 95% CI: 9.59–12.57). Among the most frequent symptoms were some level of sadness (60.14%), reduced interest in daily activities (57.97%), concerns regarding physical problems (55.79%), reduced interest in sex (52.17%), and feeling tired (51.44%). Table 2 presents the assessment results concerning sexual stigma, HIV stigma, and their respective subscales.

High scores were found on the sexual stigma scale, both internalized and perceived in the community, with slightly higher scores on the perception of social oppression. Regarding serological stigma, the total scores were high, especially on the disclosure subscale. Table 3 shows the correlations among depression, sexual stigma, HIV stigma, and adherence to ART.

The results indicate a negative, moderate, and statistically significant correlation between depression and adherence to ART (r = −0.388; *p* = 0.009), and negative, weak, and statistically significant correlations between serological stigma and adherence to ART (r = −0.181; *p* = 0.033). Although there was no correlation between sexual stigma and its subscales with ART adherence, a weak and significant positive correlation was found between sexual stigma and depression (r = 0.273; *p* < 0.001), and a moderate and significant positive correlation was found between sexual stigma and HIV stigma (r = 0.433; *p* < 0.001). Additionally, income, age at diagnosis, and education showed a weak and significant positive correlation with ART adherence. In the stepwise regression analysis, depression and age at diagnosis were variables that affected adherence to ART, explaining 20% and 22% of the outcomes, respectively. Table 4 presents the results of the regression analysis.

## 4. Discussion

Our study offers a comprehensive perspective of the complex interactions between psychosocial factors and ART adherence among MSM with HIV in Brazil. Our results revealed that approximately 20% of participants were found to have inadequate adherence to ART. Similar results were found in a Brazilian study, in which irregular adherence was observed in 25% of participants [40]. A systematic review that assessed adherence to ART in 11 Brazilian studies found insufficient or inadequate adherence between 20% and 40% in most of the studies analyzed, regardless of the assessment method [41]. Therefore, the results in terms of inadequate adherence to ART among the MSM evaluated, even with the additional burden of stigma related to sexual stigma, were not higher than those observed in other Brazilian studies with samples of people with different sexual orientations.

We found that almost 50% of the participants had some level of depression according to the Beck depression inventory-II (score > 10). This result is consistent with previous studies [20,23,42,43]. It is slightly higher than the one reported in a meta-analysis [44], in which results from 18 studies on the prevalence of depression among MSM with HIV (n = 7653) and without HIV (3395) found that depression was prevalent in 43% of HIV-positive MSM, who had a higher chance of depression compared to MSM without HIV (OR = 1.46, 1.05–2.03, *p* < 0.05).

The high prevalence of depression found in this study is consistent with minority stress theory [45], which postulates that minority groups, such as MSM, are exposed to an overload of stressors. This overload is the sum of the stressors of everyday life faced by the general population and those explicitly associated with the social impact of homophobia, such as experiences of bullying, rejection, isolation, physical or verbal violence, and even less noticeable forms of microaggression. Moreover, the systematic contact of MSM with homophobic messages throughout development may produce internalized sexual stigma, which has deleterious effects on the mental health [43,44]. According to Meyer’s (2023) [45] conceptual basis, validated by several empirical studies, individuals with minority characteristics often present with poor mental health outcomes. According to the author, if two variables that characterize the individual as a minority are added, as is the case with HIV-positive MSM, the burden of stressors and the prevalence of mental disorders increase even more.

Consistent with previous studies [21,46,47], we found a moderate and significant negative correlation between depressive symptoms and adherence to ART. Furthermore, in our regression analysis, depression was a predictor of lower ART adherence. This result corroborates the findings of Oh et al. (2023) [46], in which 601 Korean individuals living with HIV were evaluated. The authors found that participants with depressive symptoms were more likely to fail to adhere to ART therapy (adjusted OR = 0.52, 95% CI 0.34, 0.79, *p* = 0.002). Our results also confirm the findings of Paredes et al. (2024) [47], who evaluated 221 British participants living with HIV and found that inadequate adherence to ART was 72% higher among participants who tested positive on a depression screening instrument compared to those who tested negative. This finding underscores the adverse influence of depression on an individual’s ability to consistently adhere to the prescribed ART regimen, potentially compromising their motivation, energy, and perception of life. Therefore, the prevention, early identification, and effective treatment of depression are crucial for improving treatment outcomes among MSM living with HIV.

Our results indicated a high perception of sexual stigma, both internalized and in the community. These results corroborate those of previous Brazilian studies [13,48,49]. We believe that this finding may be associated, among other elements, with Catholic and Evangelical religious beliefs, which together account for more than 80% of Brazilian religions [50]. These religions propagate narratives in which homosexuality is associated with sin, immorality, promiscuity, unhappiness, and loneliness [51].

Sexual stigma was not directly correlated with adherence to ART in this study. However, its correlation with depression and HIV stigma was verified, with the latter two variables lowering adherence to ART. Therefore, our data suggest an indirect association of internalized and perceived community sexual stigma on adherence to ART, possibly mediated by depression and HIV stigma. Johnson et al. (2008) [28] also reported the possible mediating role of depression in the relationship between sexual stigma and adherence to ART. The negative correlation between HIV stigma and ART adherence suggests that individuals facing more significant HIV-related stigma may encounter additional barriers to treatment adherence [42]. These barriers include fear of discrimination, concerns about disclosing serological status, and the psychological impact of internalized stigma, all of which may undermine motivation and the ability to follow a prescribed ART regimen.

Moreover, the intersectionality of stigmatizing experiences was evident in the positive correlation between sexual stigma and depression. Both internalized and perceived sexual stigma within the community may contribute to developing depressive symptoms, perpetuating a cycle of social marginalization and mental health impairment. Thus, interventions addressing not only HIV-related stigma, but also internalized and perceived sexual stigma are essential for promoting mental health and treatment adherence among MSM living with HIV in Brazil. Since HIV-related stigma, in its different manifestations, has been identified as a threat to the global response to the HIV epidemic [14,25,26], several interventions have been developed and tested to address it. A recent systematic review, in which 70 articles were evaluated, indicated different fronts for confronting HIV-related stigma [52]. The most-tested measures aimed at treating the harmful effects associated with stigma include tailored interventions to manage internalized homophobia, which usually shows a bidirectional relationship with HIV-related stigma.

Interventions in this area mostly involve the creation of support groups for people living with HIV, respecting sociodemographic specificities and those related to other possible additional stigmas, such as sexual stigma, racism, and ageism [52]. Meetings typically occur weekly, for several months, and are led by trained facilitators or mental health professionals. Interventions comprised psychoeducational stages, in which information is presented about HIV diagnosis, treatment, possible impacts on physical and mental health, as well as strategies to minimize them. There are also psychotherapeutic approaches, usually based on cognitive-behavioral psychotherapies, associated with the identification and analysis of dysfunctional beliefs about HIV, as well as training in socioemotional skills [52]. The focus of these groups is also the prevention of social isolation, which is a common phenomenon among MSM diagnosed with HIV, and is associated with poorer mental and physical health [27,53]. Thus, these groups promote social networking and the exchange of literacy between individuals in a similar situation [52].

The occasional participation of trained lawyers to act as advocates for the rights of people living with HIV also takes place. These professionals provide information on rights guaranteed by law to people living with HIV, such as the right to HIV confidentiality, the illegality of requesting HIV serology by companies and the right to dignity in the community, as well as information on how to proceed in situations in which these legal frameworks may be disrespected [52]. Furthermore, meetings specifically arranged for the purpose of health education are also developed, in which a health professional, in addition to presenting relevant information about the dynamics of HIV infection and its effects on the body, also promotes nutritional and lifestyle counseling, with the aim of increasing the quality of life and promoting protective factors for the physical and mental health of participants. In addition to group meetings, several interventions also offer participants individual psychotherapy sessions, based on specific eligibility criteria, with the aim of covering the specificities of each case [52]. Finally, a review by Ferguson et al. (2023) [52] points to interventions aimed at health professionals involved in the care of people living with HIV. These also include the training of health education and psychoeducational groups with the aim of reducing stigma related to HIV in this population.

Therefore, although there is evidence of efficacy and effectiveness of interventions aimed at reducing HIV-related stigma and possible other associated stigmas, such as homonegativity, to the best of our knowledge, they are unformal and uncontrolled in Brazil, lacking clinical and experimental trials aiming at evaluation its effects. Therefore, we recommend developing and evaluating specific and culturally sensitive interventions for reducing stigma, supporting mental health, and encouraging treatment adherence. These interventions may include educational programs, awareness campaigns, and psychosocial support aimed at LGBTQIA+ communities and healthcare professionals. Additionally, we suggest exploring innovative approaches, such as using communication technologies and mobile applications to provide ongoing support and monitor treatment adherence. Finally, it is imperative to promote an integrated and collaborative approach among healthcare professionals, researchers, activists, and community members to effectively address these issues and improve the health outcomes of MSM living with HIV in Brazil.

Although we believe that our objectives have been achieved, we must acknowledge the study’s limitations. Our study was cross-sectional in nature, which precludes establishing causal relationships between the variables studied. It means that in our study we cannot determine the causal relationship between depression and low ART adherence, since low ART adherence may increase the risk of poor control for HIV infection, and poor physical health can exacerbate depression. In addition, possible confounders not analyzed in our study may also result in both depression and low ART adherence.

Our research comprised a non-probabilistic sample, formed by the indication of seed participants, who are mental health professionals affiliated with a Brazilian public university in São Paulo. Thus, a possible selection bias may be related to these professionals potentially recommending individuals with greater exposure of their own sexual orientation and HIV diagnosis. Hence, it is possible that individuals whose sexual orientation and HIV diagnosis have not been exposed, even to the closest social support network, may have been under-represented. Our belief, based on clinical experience and previous studies, is that in these cases, the prevalence of signs and symptoms of depression could be even higher, given that social support moderates the effects of stigma related to sexuality and HIV.

Furthermore, the majority of participants in our study were living in São Paulo, the most populous, multicultural, and developed capital of Brazil, where both homosexuality and HIV are more frequent and less stigmatized. We consider that sexual and serological stigma in this city is more openly discussed compared to less developed regions of the country, notably the north and northeast. Thus, it is possible that MSM living with HIV in the less developed regions of the country, as well as in small urban areas may face a greater burden of sexual and HIV-related stigma. Our impression is that among these individuals, the probability of encountering signs and symptoms of depression that may affect adherence to ART treatment may be higher. Therefore, these limitations suggest caution in generalizing our findings. Hence, conducting replication studies with larger and more probabilistic samples from all regions of Brazil as well as longitudinal research will be crucial to deepening our understanding of the relationship between stigma, mental health, and adherence to ART in Brazilian MSM living with HIV.

Furthermore, we use self-report instruments to assess the variables of interest. Social desirability may have influenced self-reported adherence to ART, implying an overestimation. In turn, stigma and depression may have been underestimated by the participants’ capacity for self-perception. We suggest that future studies in Brazil that compare objective measures to participants’ reports, especially regarding adherence to ART, take into consideration these possible discrepancies and their implications when conducting new research. Finally, clinical variables such as medication regimen and viral load were self-reported by participants, and not checked in their medical records. This omission can also produce a social desirability bias, mainly due to an undetectable viral load.

## 5. Conclusions

The high prevalence of depression, HIV stigma, and sexual stigma, and their adverse effects on ART adherence, points to the need of implementing evidence-based interventions to reduce sexual and serological stigma in the general population, as well as to mitigate the negative impacts of stigma on MSM living with HIV in Brazil. Stigma compromises the effectiveness of ART and increases the risk of health complications and viral transmission. Therefore, developing comprehensive strategies aimed at educating the community, combating prejudice, and promoting the acceptance of and respect for sexual and serological diversity is essential. These interventions should be tailored to the specific needs of the population of MSM living with HIV and should be implemented at different levels, including at the public policy level, in public health, and in educational programs, as well as across societal organizations. Furthermore, it is essential to conduct regular screening of depression, HIV-related stigma, and sexual stigma among MSM who receive treatment from public health services in Brazil. This practice will allow for earlier identification of mental health needs and destigmatization, enabling timely interventions and providing the necessary psychosocial support to improve the overall health, quality of life, and mental health outcomes of Brazilian MSM living with HIV.

## Figures and Tables

**Table 1:** Sociodemographic and clinical characterization of participants (n = 138).

Variable	Categories	N	%
Education Level	Incomplete Elementary School	2	1.45
	Complete Elementary School	3	2.17
	Incomplete High School	4	2.90
	Complete High School	26	18.84
	Technical Course	9	6.52
	Complete Higher Education	36	26.09
	Incomplete Higher Education	13	9.42
	Incomplete Postgraduate Studies	5	3.62
	Complete Postgraduate Studies	30	21.74
	Master’s Degree	5	3.62
	Doctorate	5	3.62
Employment Status	Employed	108	78.26
	Unemployed	21	15.22
	Retired	2	1.45
	On sick leave	7	5.07
Housing Condition	Own	64	46.38
	Rented	62	44.93
	Borrowed/Provided	12	8.70
Living Arrangements	With family/partner	80	57.97
	With colleagues/friends	12	8.70
	Alone	46	33.33
Quality of Life Post-Diagnosis	Improved	55	39.86
	Unchanged	63	45.65
	Worsened	20	14.49
Physical Health Post-Diagnosis	Improved	46	33.33
	Unchanged	63	45.65
	Worsened	29	21.01
Mental Health Post-Diagnosis	Improved	18	13.04
	Unaffected	54	39.13
	Worsened	66	47.83
Medication Used	Dolutegravir/Tenofovir/Lamivudine	100	72.46
	Other	38	27.54
Viral Load (Last Exam)	Detectable	15	10.87
	Undetectable	123	89.13

**Table 2:** Mean scores, median, standard deviation, maximum and minimum scores, and confidence intervals of the scales and subscales of sexual stigma and HIV-related stigma (n = 138).

	Possible Score	Theoretical Mean	Mean	SD	Minimum	Maximum	CI 95%
Total Sexual Stigma	19–76	47.50	49.33	3.74	38.00	61.00	48.71–49.95
Perception of Internalized Stigma	15–60	37.50	37.98	3.94	28.00	48.00	37.43–38.53
Perception of Social Oppression	4–16	10.00	11.35	1.32	8.00	16.00	11.13–11.57
Total HIV-related Stigma	40–160	100.00	98.56	21.08	50.00	157.00	95.04–102.08
Public Attitudes	20–80	50.00	49.78	10.54	20.00	80.00	48.02–51.54
Negative Self-Image	13–52	32.50	28.60	8.31	13.00	50.00	27.22–30.00
Disclosure	10–40	20.00	30.01	6.00	12.00	40.00	29.01–31.02
Personalized Stigma	18–72	45.00	38.89	11.70	18.00	72.00	36.94–40.84

Note: SD = standard deviation; CI = confidence interval.

**Table 3:** Correlations between study variables and adherence to ART (n = 138).

Variables Correlated with Adherence to ART	Correlation (Pearson)	Lower CI-95%	Upper CI-95%	*p*-Value
Depression	−0.388	−0.522	−0.237	0.009 *
Total HIV-related Stigma	−0.181	−0.338	−0.015	0.033 *
Personalized Stigma	−0.211	−0.366	−0.046	0.013 *
Disclosure	−0.066	−0.231	0.102	0.440
Negative Self-Image	−0.195	−0.351	−0.029	0.022 *
Public Attitudes	−0.169	−0.327	−0.002	0.047 *
Total Sexual Stigma	−0.106	−0.268	0.062	0.217
Perception of Internalized Stigma	−0.092	−0.256	0.076	0.281
Perception of Social Oppression	−0.066	−0.230	0.103	0.445

Note: CI = confidence interval. * *p* < 0.05.

**Table 4:** Linear regression analysis of sociodemographic variables, depression, HIV stigma, and sexual stigma (n = 138).

	*R* ^2^_adjusted_	SE	Lower CI-95%	Upper CI-95%	*p*-Value
Current Age	0.006	0.082	−0.157	0.169	0.938
Education Level	0.413	0.312	−0.205	1.031	0.188
Income	−0.001	0.202	−0.400	0.398	0.996
Age at Diagnosis	0.218	0.097	0.026	0.410	0.027
Depression (BDI-II)	−0.199	0.085	−0.368	−0.031	0.021
Total HIV-related Stigma	−0.007	0.031	−0.069	0.054	0.810
Total Sexual Stigma	0.127	0.165	−0.199	0.454	0.441

Note: SE = standard error; Lower CI-95% = lower confidence interval 95%; Upper CI-95% = upper confidence interval 95%.

## Data Availability

Data will be available upon request.
